# Nonalcoholic steatohepatitis is associated with an atherogenic lipoprotein subfraction profile

**DOI:** 10.1186/1476-511X-13-100

**Published:** 2014-06-21

**Authors:** Kathleen E Corey, Joseph Misdraji, Lou Gelrud, Hui Zheng, Raymond T Chung, Ronald M Krauss

**Affiliations:** 1 Gastrointestinal Unit, Massachusetts General Hospital, Boston, MA, USA; 2 Department of Pathology, Massachusetts General Hospital, Boston, MA, USA; 3 Department of Internal Medicine, Bon Secours Richmond Health System, Richmond, VA, USA; 4 MGH Biostatistics Center, Massachusetts General Hospital, Boston, MA, USA; 5 Harvard Medical School, Boston, MA, USA; 6 Children’s Hospital Oakland Research Institute, Oakland, USA

**Keywords:** Ion mobility analysis, Lipid subfractions, Nonalcoholic fatty liver disease, Nonalcoholic steatohepatitis

## Abstract

**Background:**

Nonalcoholic steatohepatitis (NASH) carries an increased risk of cardiovascular disease (CVD) relative to the general population. We sought to evaluate whether differences in lipoprotein subfractions in obese patients with and without NASH contributes to this difference in CVD risk.

**Findings:**

Ion mobility analysis was performed on 78 individuals with obesity undergoing weight loss surgery. All individuals had standard of care liver biopsies performed during surgery. Patients with NASH had significantly smaller peak LDL diameter (P = 0.02, 219.0 Å vs. 222.6 Å), and levels of IDL2 (P = 0.01, 104. nmol/L vs. 133.4 nmol/L) and HDL2b (P = 0.05, 676.7 nmol/L vs. 880.1 nmol/L) compared to those without NASH. NASH patients had significantly higher LDL-IVb levels than those without NASH (P = 0.02, 49.0 nmol/L vs. 37.1 nmol/L).

The inverse association of LDL peak diameter with NASH remained significant after adjustment for diabetes (P = 0.02). HDL2b levels were inversely correlated with hepatocyte ballooning and NASH and these remained significant after adjustment for diabetes (P = 0.0017 and P = 0.007, respectively). IDL2 levels were inversely correlated with NASH, hepatocyte ballooning and fibrosis stage but these were not significant after adjustment for diabetes.

**Conclusions:**

The lipoprotein subfraction profile in subjects with NASH is characterized by small peak LDL diameter, reduced HDL2b levels and elevated LDL-IVb levels. These changes may contribute to the increased CVD seen in patients with NASH.

## Introduction

Cardiovascular disease (CVD) is prevalent in individuals with nonalcoholic fatty liver disease (NAFLD). Three community-based studies from the United States and Japan have demonstrated an increased CVD risk in individuals with radiographic evidence of NAFLD when compared to those without NAFLD [1-3]. Individuals with histologically defined nonalcoholic steatohepatitis (NASH) have also been shown to have an increased CVD mortality when compared to the general population [4,5]. Thus, individuals with both radiographic NAFLD and histologically defined NASH are at increased risk for CVD and CVD-related mortality.

Multiple factors likely contribute to the increased CVD risk seen in those with NASH including hyperinsulinemia, impaired thrombolysis and alterations in lipid and lipoprotein metabolism. To date studies of lipids in NASH have been largely limited to standard lipid profiles which include total cholesterol, triglycerides, high density lipoproteins (HDL) cholesterol and a calculated, rather than direct, measurement of low density lipoprotein (LDL) cholesterol. While these parameters are accepted markers of CVD risk, it has long been recognized that these measures provide only a crude index of disease pathophysiology and recent evidence shows that additional information can be gained from measurements of lipoprotein particles and their subfractions [6-8].

Ion Mobility (IM) is an innovative method that uses gas-phase differential electrophoretic macromolecular mobility to distinguish lipoprotein particles as a function of their size and to directly and rapidly quantify their plasma levels [9,10]. We hypothesized that IM would reveal important differences in the lipoprotein subfraction profiles of individuals with obesity with and without NASH that may contribute to the increased CVD seen in NASH.

## Methods

This cohort study included consecutively enrolled patients who underwent weight loss surgery (WLS) at the Bon Secours Health System between 2010 and 2012. Subjects were assessed by a treating clinician for weight, height, BMI and co-morbidities. Diabetes mellitus was defined by a fasting glucose ≥ 126 mg/dL, HbA1C >6.5% or known diagnosis of diabetes. Hypertension was defined by a blood pressure ≥135/85 or undergoing treatment for hypertension. Individuals on lipid lowering therapy were excluded. No individual experienced significant weight loss prior to surgery.

All subjects undergoing WLS had a standard of care liver biopsy at the time of surgery. Liver biopsies were reviewed by single blinded hepatopathologist (JM) and assigned a score for grade of steatosis (grade 0 = <5% steatosis; 1 = 5-33%; 2 = 33-66%; 3 = >66%), hepatocyte ballooning (0 = no ballooning; 1 = few; 2 = many), and lobular inflammation per 200 × (0 = no foci; 1 = <2 foci; 2 = 2–4 foci; 3 = >4 foci), as described by Kleiner et al. [11] NAFLD activity score (NAS) is a sum of the scores for steatosis grade, lobular inflammation and hepatocyte ballooning and ranges from 0–8. Fibrosis stage was assigned according to the modified Brunt stage (stage 0, 1a, 1b, 1c, 2, 3, or 4) [11]. Non-NASH was defined by the presence of grade 0 or greater not meeting criteria for NASH. NASH was defined as lobular inflammation > =1, hepatocyte ballooning > =1 and steatosis grade > =1 [12].

### Ion mobility analysis

IM was used on to directly quantify the full spectrum of lipoprotein particles, from the smallest, densest HDL particles to large buoyant VLDL particles, in baseline plasma samples as described previously.

### Statistical analysis

All statistical analyses were performed using SAS software, version V.9.2 (SAS Institute, Cary, NC). Continuous variables were analyzed using a Student’s *t*-test for normally distributed variables and a Wilcoxon rank sum test for variables that were not normally distributed. Categorical variables were analyzed using a Chi square test or Fisher’s exact test as appropriate. Multivariable logistic regression modeling was used to assess the independent association of OSA with histology.

This study was approved by the Partners’ Health Care Human Research Committee.

## Results

### Baseline characteristics

Seventy-eight individuals were included in this cohort. Mean age was 45.6 years and mean BMI was 47.3 kg/m^2^. Eighty-five percent of the patients were women, 78.2% were white and 35.9% carried a diagnosis of diabetes mellitus.

### Clinical characteristics of NASH and non-NASH patients

There were no differences in mean age or BMI in NASH and non-NASH patients (Table 1). Diabetes mellitus and hyperlipidemia were more prevalent in NASH patients than in non-NASH patients (P = 0.03, 50.0% vs. 25% and P = 0.02, 54.1% vs. 26.9%, respectively). Mean HDL cholesterol was lower in patients with NASH when compared to those without NASH (P = 0.003, 36.5 vs. 41.8 mg/dL). Both LDL cholesterol and total cholesterol were higher in non-NASH patients (P = 0.007 and 0.009, respectively). There were no significant differences in insulin or triglyceride levels.

**Table 1:** Baseline characteristics

	**Non-NASH**	**Definite NASH**	**P Value**
**Mean age in years (SD)**	44.1 (11.9)	47.3 (13.3)	0.27
**Gender, n (%)**			
**Female**	37 (90.2)	28 (79.7)	0.12
**Race, n (%)**			
**Black**	13 (31.7)	4 (10.8)	
**White**	28 (68.3)	33 (89.2)	0.03
**Mean BMI (SD)**	46.8 (6.4)	48.0 (7.3)	0.43
**Obstructive sleep apnea, n (%)**	23 (56.1)	26 (67.6)	0.36
**Diabetes mellitus, n (%)**	10 (25.0)	18 (50.0)	0.03
**Mean insulin, μU/dL(SD)**	32.4 (27.3)	32.3 (19.1)	0.98
**Mean high density lipoprotein, mg/dL (SD)**	41.8 (7.9)	36.5 (7.5)	0.003
**Mean triglyceride level, mg/dL (SD)**	108.9 (69.9)	122.1 (73.0)	0.42
**Mean low density lipoprotein, mg/dL (SD)**	101.4 (32.9)	81.7 (29.2)	0.007
**Mean total cholesterol, mg/dL (SD)**	165.0 (40.6)	142.8 (31.7)	0.009

### Lipoprotein particles in NASH and non-NASH patients

LDL peak diameter was significantly smaller in NASH patients those without NASH (P = 0.02, 219.0 Å vs. 222.6 Å) (Table 2). Notably, LDL peak diameter was inversely correlated with hepatocyte ballooning (P = 0.007), steatosis grade (P = 0.022) and NAS (P = 0.015). When adjusted for the presence of diabetes the inverse correlation between LDL peak diameter and NAS remained significant (P = 0.02) while the significance of the correlations for hepatocyte ballooning and steatosis grade was reduced (both P = 0.06).

**Table 2:** Lipoprotein subfraction profiles in those with and without NASH

	**Non-NASH**	**Definite NASH**	**P Value**
**LDL Peak Diameter, Å (SD)**	222.6 (5.4)	219.0 (4.5)	0.02
**LDL-IVb, nmol/L (SD)**	37.1 (16.3)	49.0 (28.0)	0.02
**HDL2b, nmol/L (SD)**	880.1 (465.2)	676.6 (456.7)	0.05
**IDL2, nmol/L (SD)**	133.4 (60.8)	104.1 (38.6)	0.01

In addition, very small LDL-IVb levels were significantly higher in NASH patients than non-NASH patients (P = 0.02, 49.0 nmol/L vs. 37.1 nmol/L), and were directly correlated with hepatocyte ballooning (P = 0.03), steatosis grade (P = 0.03) and NAS (P = 0.04). The correlation with NASH remained significant after adjustment for diabetes (P = 0.02).

NASH was also characterized by low HDL2b levels when compared to non-NASH (P = 0.05, 676.7 nmol/L vs. 880.1 nmol/L). HDL2b levels were inversely correlated to hepatocyte ballooning and NAS which remained significant after adjustment for diabetes (P = 0.017 and P = 0.007).

Mean IDL2 level was significantly lower in NASH patients than non-NASH patients (P = 0.01. 104.1 nmol/L vs. 133.4 nmol/L), and IDL2 levels were inversely correlated with hepatocyte ballooning (P = 0.01), NAS (P = 0.05) and fibrosis stage (P = 0.05). However, after adjustment for diabetes these correlations were not significant.

### Lipoprotein particles in NASH and Non-NASH patient without diabetes mellitus

When this analysis was restricted to individuals without diabetes mellitus, LDL-IVb remained significantly higher in those with NASH (p = 0.03) and IDL2 remained lower (p = 0.03). LDL peak diameter and HDL2 remained lower in NASH patients but these differences were not statistically significant.

## Conclusions

The present study reveals several important novel differences in the lipid profiles on patients with and without NASH. Patients with NASH were found to have a significantly smaller LDL peak diameter than those without NASH. In addition, we found that LDL-IVb was significantly higher in those with NASH. LDL-IVb is the smallest subclass of LDL particles with a diameter of 22.0-23.3 nm. LDL-IVb represents a relatively minor subfraction of LDL, accounting for approximately 10% of LDL particles. However, a study by Williams et al. found that LDL-IVb was the single best predictor among lipoproteins of increased coronary artery stenosis [13]. Stenosis change was six times more rapid among individuals with LDL-IVb levels in the highest quartile than those with the lowest LDL-IVb levels. This study suggested that a considerable portion of CVD risk attributed to small dense LDL may be driven by very small LDL particles. Thus, our finding of increased LDL-IVb levels in NASH patients may contribute to the increased CVD experienced by this group.

HDL is composed of 5 subclasses including the small in size HDL3a, HDL3b, HDL3c, as well as the large HDL2a and HDL2b [13]. Decreased HDL2b content relative to HDL3b and HDL3c is associated with an increased CVD risk [14,15]. We found that, in individuals with NASH, HDL2b was significantly lower in those without NASH and it is possible that this is related to the increased CVD seen in NASH.

Several important limitations in the present study must be acknowledged. Our group included all individuals undergoing weight loss surgery and thus the findings may not be generalizable to the full spectrum of NAFLD and NASH patients. Finally, our study is limited by small size and predominance of white women. Future studies of increased size and diversity as well as evaluation of lipoprotein particle differences in patients with normal liver histology compared to those with steatosis and NASH are needed.

In conclusion, the present study suggests that individuals with NASH may have a more atherogenic lipoprotein subfraction profile than those without fatty liver disease.

## Competing interests

The authors declare that they have no competing interests.

## Authors’ contributions

KEC: study design, data acquisition, analysis and interpretation of data, statistical analysis, study supervision, drafting of manuscript, revision of manuscript, important intellectual content. RMK: study design, data acquisition, analysis and interpretation of data, statistical analysis, study supervision, drafting of manuscript, revision of manuscript, important intellectual content. RTC: analysis and interpretation of data, study supervision, revision of manuscript, important intellectual content. AM: analysis and interpretation of data, study supervision, revision of manuscript, important intellectual content. JM: analysis, important intellectual content, revision of manuscript. HZ: study design, analysis and interpretation of data, statistical analysis, revision of manuscript. LG: study design, revision of manuscript, important intellectual content. All authors read and approved the final manuscript.

## Main messages

• Nonalcoholic steatohepatitis (NASH) is associated with atherogenic dyslipidemia.

• The dyslipidemia of NASH is characterized by small peak LDL diameter and elevated levels of LDL-IVb.

• NASH is also associated with low levels of IDL2 and HDL2.

• These differences may contribute to the increased cardiovascular disease prevalence seen in individuals with NASH.

## Research questions

• Does the dyslipidemia result from NASH or contribute to the development of NASH?

• Does the atherogenic dyslipidemia seen with NASH improve with NASH treatment?

• How does therapy with lipid lowering agents impact this dyslipidemia
